# EMG-Triggered Functional Electrical Stimulation for Central Facial Palsy Following Stroke: A Clinical Case Report

**DOI:** 10.3390/brainsci15040410

**Published:** 2025-04-17

**Authors:** Frauke Johannes, Anna Maria Pekacka-Egli, Simone Köhler, Andreas Disko, Jan von Meyenburg, Bartosz Bujan

**Affiliations:** 1 Department for Neurorehabilitation, Zurich Rehabilitation Center Lengg, 8008 Zurich, Switzerland; annamaria.pekacka-egli@kliniklengg.ch (A.M.P.-E.);; 2 School of Health Professions, Bern University of Applied Science, 3007 Bern, Switzerland; 3 Department of Communicative Participation and Speech and Language Therapy, FHNW University of Applied Sciences and Arts, 4132 Muttenz, Switzerland

**Keywords:** FES, NMES, central facial palsy, functional electrical stimulation, EMG-triggered, rehabilitation

## Abstract

**Background:** Central facial palsy (CFP) is a common condition following stroke, typically affecting the lower face and causing symptoms such as drooling, dysarthria, and facial asymmetry. Despite available rehabilitation methods, the evidence supporting their effectiveness is limited. Electromyography (EMG)-triggered Functional Electrical Stimulation (FES) has shown promise in neurorehabilitation for motor impairments, but its application to CFP remains unclear. **Methods:** This case report explores the use of EMG-triggered FES in a 77-year-old patient with CFP following a severe ischemic stroke of the middle cerebral artery (MCA). Therapy, focused on stimulating the orbicularis oris muscle to address persistent drooling and improve facial symmetry, was alongside usual care. The stimulation duration was 5–15 min, frequency 35 Hz, and pulse duration 300 µs, applied 5 times a week. Stimulation duration was adjusted based on the patient’s progress. **Results:** The patient underwent 16 sessions of EMG-triggered FES over four weeks. Post-therapy reassessment with the Sunnybrook Facial Grading System (SFGS) showed an improvement in facial motor function, with the score increasing from 58/100 to 78/100. Reassessment of the Facial Disability Index (FDI) revealed significant improvement in physical function (55 to 85 points), though the social function score slightly decreased (76 to 64 points). Improvements in dysarthria and the complete resolution of drooling were reflected in the physical function domain of the FDI and the Allensbach Dysarthria Severity Scale. **Conclusions:** The results highlight that EMG-triggered FES was well tolerated and effectively supported therapy, contributing to the resolution of drooling, improved facial symmetry, and enhanced speech function. Future research should focus on randomized controlled trials to confirm its effectiveness and determine optimal therapy parameters.

## 1. Introduction

Central facial paresis (CFP) is a frequently observed initial presentation in stroke patients, with an estimated prevalence of 45% [1]. It is characterized by unilateral motor dysfunction contralateral to the lesion, predominantly affecting the lower face [2]. These deficits may present as weakness of the facial muscles, predominantly involving the oral and cheek musculature. As a result, the corner of the mouth and the cheek may droop, leading to facial asymmetry. This muscle weakness can also cause drooling at rest or during oral intake, along with reduced chewing strength and efficiency, as well as dysarthria [3]. Beyond these functional impairments, the condition may also result in visible aesthetic changes and is strongly associated with a considerable decline in quality of life [3,4,5]. While a variety of therapeutic approaches are available for the treatment of CFP, the evidence supporting their effectiveness remains limited [3].

Treatment approaches for facial palsy have evolved to combine traditional physiotherapy and speech therapy with advanced technologies. Biofeedback and neuromuscular electrical stimulation (NMES) support muscle re-education, while newer methods like mirror therapy, virtual reality, and transcranial direct current stimulation (tDCS) aim to enhance neuroplasticity and functional recovery. Although evidence is still emerging, integrating these modalities shows promise in improving outcomes [6]. One potential rehabilitation approach is electrical stimulation, which has been used in neurorehabilitation for motor recovery [7]. However, the terminology surrounding electrical stimulation remains inconsistent, with terms such as Functional Electrical Stimulation (FES) and NMES often used interchangeably. In this article, we adopt the definitions provided by Schick et al. to distinguish clearly between FES as functionally oriented and NMES as predominantly passive [8]. According to their definition, FES is a subtype of electrotherapy in which the stimulation assists functional movements, whereas NMES is usually a passive form of electrotherapy that does not involve active or function-oriented patient participation [8]. Another form is Electromyography (EMG)-triggered FES, often referred to as EMG-NMES in the literature. In this method, the patient’s weak movement impulse is amplified by electrical stimulation, triggered directly by their own voluntary EMG signals. Once a predefined threshold of muscle activity is reached, the electrical stimulation takes place. In this therapy approach, some principles of motor learning, such as the repetition of movements [9], are already integrated, while others can be easily incorporated to further support the motor learning process.

In recent years, FES has gained recognition as a promising intervention for enhancing various functions in neurorehabilitation, such as arm and hand function and has demonstrated potential superiority over other stimulation methods within comprehensive rehabilitation treatment [10,11]. While there is already literature on the use of FES in peripheral facial palsy, indicating potential benefits for facial muscle rehabilitation [12,13], research on the application of electrotherapy for CFP remains sparse. Some studies have explored its effects on facial muscles in the context of dysphagia therapy, but the available evidence is still limited. For instance, Lee et al. compared the application of NMES in both the masseter and suprahyoid muscles with stimulation of the suprahyoid muscles alone [14]. The results, assessed using the American Speech-Language-Hearing Association National Outcome Measurement System (ASHA-NOMS), the Functional Dysphagia Scale, and the Penetration Aspiration Scale, did not show significant differences between the groups. In addition, studies by Choi et al., which focused on stimulating the paretic facial region [15], and Oh et al., who targeted the orbicularis oris muscle, both reported significant improvements in swallowing function as well as in the strength of the cheeks and lips [16].

Despite these findings, none specifically explored the application of EMG-triggered FES for facial muscles and its effects on functional recovery, especially in the context of CFP. This clinical case report documents the course and results of EMG-triggered FES, with a focus on stimulating the orbicularis oris muscle, in a patient with CFP following an ischemic stroke. The aim is to highlight the practical implementation of this combined intervention and its potential impact on recovery.

## 2. Case Presentation

Before admission to our inpatient neurological rehabilitation clinic, the 77-year-old patient initially presented to the emergency department of a general hospital with dysarthria and mild left-sided hemiparesis, with an arm predominance. An ischemic infarction of the right hemisphere due to vessel occlusion of the right M2 segment of the middle cerebral artery (MCA) was identified. The initial NIHSS (National Institutes of Health Stroke Scale) [17] score was 5. Due to the time window of more than 5 h after symptom onset and the existing infarction demarcation, systemic intravenous thrombolysis was not performed. However, given the presence of a perfusion deficit with mismatch, a mechanical thrombectomy was attempted but remained unsuccessful despite multiple recanalization efforts. During her stay, her clinical condition worsened, with increased somnolence, a significantly more pronounced left-sided hemiparesis, severe dysarthria, dysphagia and multimodal neglect on the left (NIHSS 13). Imaging revealed multiple ischemic lesions throughout the entire right MCA territory, with cortical predominance, as well as extensive infarctions in the caudate nucleus, basal ganglia, and frontal operculum. A persistent occlusion of the proximal M2 (fronto-opercular artery) on the right, along with severe stenosis and remaining thrombus in the inferior trunk of the MCA on the right, was noted. A definitive stroke etiology could not be determined, although an embolic etiology is likely. For secondary prophylaxis, the patient received antiplatelet agents, statins, and antihypertensive therapy, and further diagnostics were conducted to search for a proximal embolic source. Her clinical symptoms improved during her hospitalization, and she was subsequently transferred to our inpatient neurological rehabilitation clinic. At discharge, she presented with the following symptoms:

1. Severe spastic left-sided sensomotoric hemiparesis with arm predominance
2. Central facial palsy, Fisch score [18] 76/100, left-sided
3. Dysarthria
4. Multimodal left-sided neglect
5. Mild reduction in vigilance

## 3. Investigation

After admission to our inpatient rehabilitation center, the patient underwent initial nursing and medical assessment. She was then enrolled for multimodal multidisciplinary neurological rehabilitation, including speech and language therapy (SLT), physiotherapy, neuropsychology, and occupational therapy.

NIHSS score at the beginning of rehabilitation was 14, with a facial motor subscore of 2. Her Functional Independence Measure (FIM) [19] score was 14/91 (motor subscale), 15/35 (cognition subscale), and 29/126 (total score). 

### Initial Speech and Language Therapy Assessment

During the initial speech and language therapy (SLT) the patient reported increased effort when speaking, ineffective drooling management, painful swallowing, and a frequent urge to cough. Dysphagia risk was assessed using the Standardized Swallowing Assessment (SSA) [20], which yielded aspiration predictors. A Clinical Swallowing Evaluation (CSE) [21] and flexible endoscopic evaluation of swallowing (FEES) [22] were performed, revealing no relevant signs of dysphagia. However, due to insufficient oral intake related to the patient’s overall condition, as well as the absence of teeth and prosthetic dental support, a nasogastric tube was placed for nutritional support. In the meantime, the patient was provided with a modified diet under mealtime supervision by nursing staff.

Further examination confirmed dysarthria accompanied by facial paresis. Spontaneous speech was markedly slowed and characterized by imprecise articulation. Prosody appeared monotonous, and overall speech rate was reduced. According to the Allensbach Dysarthria Severity Scale [23], the dysarthria was classified as moderate.

Facial nerve function was evaluated using the Sunnybrook Facial Grading System (SFGS) [24]. The results revealed significant asymmetry in facial movements, both at rest and during voluntary actions. At rest, eye symmetry was preserved, but the nasolabial fold was minimally pronounced, and the mouth corner appeared drooped. During voluntary movements, the patient achieved full eyebrow movement and eye closure. However, she was unable to smile with her mouth open. When showing her teeth, the movement was executed with minimal range. Lip pursing was partially possible but restricted in amplitude. The most pronounced asymmetries were observed in the zygomaticus and risorius muscles, indicating significant impairment. Severe asymmetry was also noted in the levator labii muscle, while moderate asymmetry was present in the orbicularis oris. These findings reflect considerable dysfunction of the lower facial muscles, resulting in noticeable limitations in facial expressions and movements. The SFGS yielded a score of 58/100, indicating moderate paresis. To assess the patient’s perceived disability and quality of life related to facial palsy, the German version of the Facial Disability Index (FDI) [25] was administered. The patient scored 55/100 points on the physical function subscale and 76/100 points on the social function subscale.

Overall, the findings indicate a complex clinical presentation characterized by dysarthria and facial paresis, affecting articulation, phonation, respiration, and oral-motor coordination. Additionally, pronounced visual neglect, cognitive slowing, and increased fatigability further contribute to the patient’s increased speaking effort and communication challenges.

## 4. Treatment Protocol

The treatment protocol was provided based on the clinical findings of the patient. Therapy focused on targeted stimulation of the left orbicularis oris muscle to improve lip closure, reduce drooling, promote more symmetrical facial expressions, and ultimately improve overall quality of life.

### 4.1. Contraindications

EMG-triggered FES should not be used in patients with implanted pacemakers, cardiac arrhythmias, status epilepticus, open wounds in the application area, tumors at the stimulation site, or during pregnancy. These contraindications were carefully assessed before initiating therapy to prevent complications.

### 4.2. Speech and Language Therapy

SLT was conducted twice per week, with each session lasting between 30 and 45 min. The therapy focused on facial palsy rehabilitation based on neuromuscular retraining [26] and dysarthria treatment.

### 4.3. General Considerations

**Figure 1:**
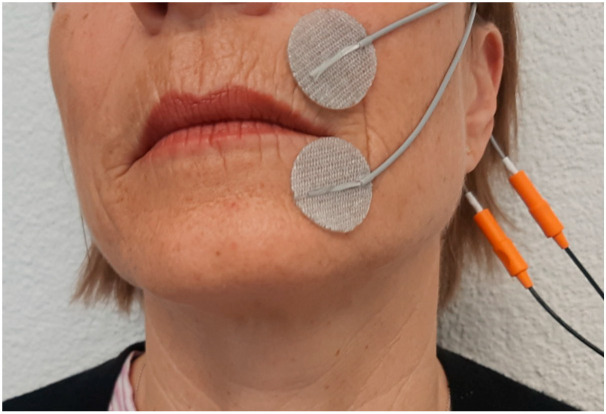
Position of the electrodes for stimulation of the orbicularis oris muscle—demonstration picture.

To ensure safety and effectiveness, a minimum of six hours was maintained between EMG-triggered FES sessions. The electrodes used for stimulation had a diameter of 2.5 cm and were applied as shown in Figure 1. The timing of the sessions was flexible and adjusted according to the patient’s needs. Prior to each session, the patient was informed about potential sensations such as a metallic taste or brief flashes of light.

For the therapy, the patient was positioned in an upright seated position with a supportive surface for her arms to ensure stability and comfort.

The treatment regimen initially aimed for five sessions per week, with each session lasting up to 20–30 min [27]. However, this frequency was not always maintained, and both the duration and frequency of treatment varied. The stimulation duration ranged from 5 to 15 min, depending on the patient’s attention span and occasional fatigue, which required adjustments, sometimes resulting in shorter sessions or rescheduled appointments.

### 4.4. Device Settings

The STIWELL^®^ PROFES electrostimulation device was used. EMG-triggered FES was administered using standardized parameters tailored to individual tolerance levels, following the program settings and recommendations provided by the device manufacturer. Initially, therapy was conducted using a basic EMG-triggered program aimed at facial muscle activation while the patient was also instructed to perform a blowing action to trigger the stimulation. However, after one week, therapy was switched to a different program due to persistent attentional deficits and neglect-related challenges. The same parameters were transferred to this new program, which introduced a visual feedback component to enhance the patient’s engagement.

In this new program, the screen of the device displayed an airplane that the patient could set into motion by activating the orbicularis oris muscle. To achieve this, the patient was instructed to perform a puffing motion with the lips, activating the orbicularis oris muscle. The task was to ‘blow the airplane away’, providing direct visual feedback on the movement initiation and reinforcing correct muscle activation. Additionally, the program incorporated background noise resembling the sounds of an airport terminal, including audio effects corresponding to an airplane takeoff, which further aided in capturing the patient’s attention and encouraging engagement. The screen was positioned to ensure effortless visual exploration, accommodating the patient’s neglect and facilitating interaction with the task.

This change significantly improved the patient’s compliance and increased the frequency of successful triggers over time. In both programs, if the patient experienced dysesthesia or discomfort during stimulation, the amplitude was adjusted accordingly to ensure a tolerable and effective treatment intensity.

The following stimulation parameters were used, with the threshold continuously adjusted throughout the therapy course to optimize the treatment based on the patient’s progress.

- Intensity: 6–8 mA
- Threshold Levels: 60–100%, depending on calibration and impedance
- Stimulation Duration: 5 to 15 min
- Frequency: 35 Hz
- Pulse Duration: 300 µs
- Ramp-Up Time: 0.5 s
- Plateau Time: 3 s
- Ramp-Down Time: 0.5 s
- Pause Time: 5 s

## 5. Follow-Up and Outcomes

The patient underwent EMG-triggered FES five days per week (Monday through Friday) during four weeks, totaling 16 sessions. A follow-up evaluation using the SFGS was conducted after four weeks. Facial symmetry at rest remained unchanged compared to baseline. During voluntary movement, the patient achieved full eyebrow elevation and complete eye closure. Smiling with an open mouth was possible but remained restricted in range. The patient demonstrated full movement when displaying her teeth, and lip pursing was nearly complete. Muscle asymmetries were noted as follows: moderate asymmetry in the zygomaticus and risorius muscles, indicating improvement relative to baseline; mild asymmetry in the levator labii and orbicularis oris muscles. The SFGS yielded a score of 78/100, consistent with mild residual paresis. These findings suggest progressive recovery of facial motor function, with reduced asymmetry severity compared to the initial assessment. SFGS-assessed outcomes comparison is presented in Table 1.

Reassessment of the FDI yielded 84/100 points on the physical function subscale and 64/100 points on the social function subscale. A comparison of FDI-assessed outcomes is presented in Table 2.

Swallowing function was also reassessed, leading to a recommendation for nasogastric tube removal and dietary progression to solid food due to absence of teeth and prosthetic dental support.

Despite persistent mild facial paresis, spontaneous speech demonstrated improvement, characterized by increased articulatory precision and a modest acceleration in speech rate. Prosodic modulation exhibited greater variability and according to the Allensbach Dysarthria Severity Scale, dysarthria was now classified as mild to moderate. Moreover, there was a complete resolution of drooling.

### 5.1. Adverse and Unanticipated Events

There were no adverse or unanticipated events related to FES treatment. Visual inspection of the face after each session did not raise any concerns regarding skin and tissue irritation due to stimulation.

### 5.2. Remaining Rehabilitation Stay and Discharge from Rehabilitation

FES therapy was continued until discharge. The patient remained in the facility for a total of 39 days, taking part in a multidisciplinary rehabilitation to reinforce therapeutic gains. At discharge, the Functional Independence Measure (FIM) scores were 24/91 (motor subscale), 23/35 (cognitive subscale), and 47/126 (total score). The NIHSS score was 12, with a facial motor subscore of 2, and a pronounced visual neglect remained. Improvements in facial nerve function, as reflected in both FDI and SFGS scores, were observed, with enhanced facial muscle mobility and increased symmetry during voluntary and spontaneous movements. The patient was discharged to a care home.

*a*. *Clinical Data Collection*

All clinical data were collected as part of routine inpatient care by the interdisciplinary rehabilitation team. Standardized assessments such as the SFGS, the FDI and FIM were performed at admission and discharge. The collected data were documented in the clinic’s electronic health records and used retrospectively for this case report with the patient’s informed consent.

## 6. Discussion

The aim of this Case report was to highlight the practical implementation of EMG-triggered FES and its potential impact on recovery in CFP, since this has not been reported yet. It describes the use of EMG-triggered FES as an adjunct therapy within the usual care of inpatient neurorehabilitation with CFP following a severe stroke.

In this case report, we focused on the stimulation of the orbicularis oris muscle to specifically target the persistent drooling, which was highly distressing for the patient. Over the course of treatment, the patient showed significant improvements, including a notable functional increase in the SFGS score. The subjective impression of improved dysarthria and the complete resolution of drooling were consistent with the reported scores in the physical function domain of the FDI and the improvement in dysarthria observed in the Allensbach Dysarthria Severity Scale. Facial expressions are a key component of social interaction. Mimicry, the often-unconscious imitation of another person’s facial expressions, plays a subtle but important role in social communication and mutual understanding [28]. In the context of CFP, impairments in facial expressiveness may therefore affect the quality of social interactions [3,29]. While improvements in motor function were observed in our case, these did not appear to translate into a corresponding improvement in perceived social participation or quality of life.

Notably, not all aspects of social functioning were rated as impaired. For instance, Item 10 of the FDI social function domain (“How often has your facial function kept you from going out to eat, shop, or participate in family or social activities?”) was consistently rated with the highest possible score (“none of the time”). This suggests that while participation in everyday activities was not restricted, more nuanced aspects of social-emotional interaction may have remained challenging. In addition, throughout the inpatient stay, the patient’s emotional well-being fluctuated as she adapted to her new life situation and increasing dependence on assistance. Symptoms suggestive of post-stroke depression, which is common after stroke [30], may also have influenced her perception of social functioning and contributed to the low score in this domain.

These observations highlight the intricate interrelationship between motor recovery, emotional adjustment, and social reintegration in individuals with CFP. Although measurable functional improvements can be achieved, these do not necessarily translate into enhanced perceived social participation, emphasizing the need for comprehensive and patient-tailored rehabilitation approaches that address both motor and psychosocial dimensions of recovery.

Despite its clinical relevance, the course of CFP following stroke remains insufficiently documented. The frequently cited study by Svensson et al. included only 35 patients and reported significant spontaneous improvement within one month, with two-thirds of patients showing normal or mild dysfunction after six months [31]. However, a more recent study by Volk et al., which evaluated 112 inpatients in a rehabilitation setting and assessed both motor and non-motor impairments, reported that even at discharge —after a median of 41 days—60% of patients still had a House–Brackmann grade ≥ III [32,33]. This suggests that recovery of CFP can be prolonged and incomplete, raising interest in therapeutic approaches that actively promote neuroplasticity, especially in the early stage after a central lesion. Current treatments, including facial exercise therapy and NMES, show mixed results, with recovery varying based on timing and patient factors. Emerging technologies like home-based telerehabilitation and wearable devices offer promising alternatives, particularly for early-stage rehabilitation. Future research should compare these treatments more directly and integrate newer technologies to strengthen evidence-based recommendations for CFP management.

### 6.1. Scientific Rationale

FES activates nerve-fibers through an electric field between two surface electrodes. This field depolarizes nearby neurons; if the threshold is exceeded, an action potential is triggered, propagating along the axon to the motor endplate, causing muscle contraction [31]. With the parameters used, muscles are not directly stimulated, as motor nerves have a significantly lower excitation threshold than muscle cells [34]. The therapeutic effects of FES are thought to result from the activation of sensory and motor areas, promoting cortical plasticity and facilitating the reintegration of sensorimotor pathways through corticospinal adaptation [35,36]. In addition to these mechanisms, EMG-triggered FES inherently supports training-induced plasticity [37,38], combining voluntary movement modulation, proprioceptive sensory feedback, and electrical stimulation.

Given the severity and complexity of our patient’s impairment, we assume that these key factors played a crucial role in the successful implementation of the therapy. In particular, the integration of motor learning principles appears to have been essential in facilitating progress.

### 6.2. Motor Learning

The early decision to switch to a program with visual feedback seemed to offer a promising approach, as it aligned with several principles of motor learning. Before the change, the patient required significant support to sustain the movement until the stimulation took place. Maintaining activation of the orbicularis oris muscle was challenging, and her engagement in the task was inconsistent. Often, the movement was prematurely abandoned before the stimulation could be activated. After switching to the program with visual feedback, the patient demonstrated noticeable progress: the triggers for the stimulation became more frequent, and she required less support. Additionally, the duration of the stimulation time increased, and the change in the program also led to increased motivation, with the patient showing greater engagement and more consistent participation in the therapy. Several principles of motor learning are addressed through the use of EMG-triggered FES, which may have played a role in the patient’s progress.

Motivation plays a crucial role in motor learning, as using external focus, like the airplane on the screen, helps shift attention from isolated muscle activation to a more task-oriented movement [39,40]. This reduces self-focus and enhances motivation by providing positive movement experiences with direct visual feedback [39,40]. The game-like character of this approach further enhances engagement by making the exercise more interactive and enjoyable while the airplane serves as a clear indicator of whether the movement is successful and reinforcing correct activation patterns. Additionally, the stimulation itself can serve as a tactile cue to support movement execution, further supporting motor learning. Auditory cues can also help to redirect the patient’s attention back to the task, which is particularly beneficial for individuals with attention deficits.

Frequent repetition is another important factor in motor learning, as it helps stimulate plastic changes [41,42]. To ensure that the movements do not become monotonous and remain effective, they should be gradually varied over time [43]. This can be achieved through shaping, which involves the progressive adjustment of the activity to consistently challenge the patient at their current performance limit [43]. In EMG-triggered FES, shaping can be achieved by adjusting exercise conditions, the task itself, or various time parameters. A key advantage of this approach is the immediate feedback on task difficulty, as the patient can set the activation threshold based on their achievable muscle activity, allowing adjustments to be made to maintain an optimal balance between challenge and achievability, as recommended [44].

### 6.3. Strengths and Limitations

This report is limited in that case reports provide low-level evidence in medical literature [45], and further studies with larger sample sizes are necessary to draw broader conclusions. Additionally, it must be taken into account that the assessments used to represent the progression of CFP in this case, especially the SFGS, was developed for peripheral facial palsy and has not been validated for CFP. The SFGS may potentially underestimate the progress of patients with primarily lower face involvement and without synkinesis, as is the case in CFP. Due to the lack of validated assessments for CFP, these were the most appropriate tools available for evaluation. The NIHSS facial subscore specifically evaluates the ‘show teeth’ function and is not designed to differentiate or adequately assess facial palsy, nor does it capture the function of the orbicularis oris muscle.

The strengths of this report include the comprehensive treatment protocol for EMG-triggered FES, as detailed in this report, demonstrates not only the safety and effectiveness of this approach but also highlights its practical applicability in a clinical setting. The data further support the clinical reasoning behind the use of EMG-triggered FES in treating CFP, underlining its potential benefits in facilitating patient progress while ensuring safety and optimizing outcomes.

## 7. Conclusions

This case report presents the use of EMG-triggered FES in a patient with CFP following a severe stroke, aiming to highlight its practical implementation and potential impact on recovery. The report demonstrates that the treatment was easily integrated into therapy, well received by the patient, and supports the safety and tolerability of electrical stimulation in this mode of application. Without EMG-triggered FES, performing the exercises would have been significantly more challenging and frustrating for the patient, potentially hindering her progress. Future research should include randomized controlled trials to generate high-level evidence on the effectiveness of EMG-triggered FES for improving facial function in post-stroke patients, particularly in those where spontaneous remission is unlikely. The design of such trials should address both the direct impact on the paralyzed facial muscles and the most appropriate application parameters.

## 8. Patient Perspective

After the process was explained to the patient, including that the additional stimulation with EMG-triggered FES would be used to address her drooling, the patient immediately agreed to further participate in the treatment. FES is typically associated with sensory side effects, and this patient also experienced them. Nevertheless, she looked forward to the stimulation sessions, despite occasionally feeling some discomfort, particularly under the electrode. After the program was changed, she engaged with it enthusiastically, saying, “Today, we’re sending planes into the desert again”. She perceived it as a welcome variation from her usual therapy, and this playful framing seemed to enhance her motivation and engagement in therapy. Over the course of the treatment, the drooling completely disappeared, which the patient reported with satisfaction.

## Figures and Tables

**Table 1:** Assessments of the Sunnybrook Facial Grading System.

	Before	After
**Synkinesis Score**	0	0
**Resting Symmetry**		
Eye	1	1
Cheek	0	0
Mouth	1	1
**Symmetry of voluntary movements**		
Forehead wrinkle	5	5
Gentle eyes closure	5	5
Open mouth smile	2	3
Snarl	2	5
Lip pucker	3	4

Scores at admission and after 4 weeks of treatment; Resting Symmetry: 0—Normal, 1–2—Asymmetric; Symmetry of Voluntary Movements: 1—No movement, 2—Slight movement, 3—Mild excursion, 4—Movement almost complete, 5—Complete movement; Synkinesis Score: 0—None, 1—Mild, 2—Moderate, 3—Severe.

**Table 2:** Assessments of the Facial Disability Index.

	Before	After
Physical function	55	85
Social function	76	64

Scores at admission and after 4 weeks of treatment; Physical and Social function Score 0–100 each, where 0—worst, 100—best.

## Data Availability

The data presented in this study are available in this article.
